# Factors associated with 30-day mortality in patients with acute heart failure presenting to the emergency department: a retrospective cohort study

**DOI:** 10.1186/s12872-025-05430-z

**Published:** 2025-12-12

**Authors:** Chanon Changratanakorn, Apichat Tantraworasin, Jiraporn Khorana, Borwon Wittayachamnankul

**Affiliations:** 1 Emergency Medicine Department, Faculty of Medicine, Chiang Mai University, 110 Inthawaroros Road, Sribhumi, Amphoe Muang, Chiang Mai, 50200 Thailand; 2 Division of Thoracic Surgery, Department of Surgery, Faculty of Medicine, Chiang Mai University, Chiang Mai, Thailand; 3 Clinical Surgical Research Center, Faculty of Medicine, Chiang Mai University, Chiang Mai, Thailand; 4 Clinical Epidemiology and Statistical Statistic Center, Faculty of Medicine, Chiang Mai University, Chiang Mai, Thailand; 5 Department of Biomedical Informatics and Clinical Epidemiology, Faculty of Medicine, Chiang Mai University, Chiang Mai, Thailand; 6 Division of Paediatric Surgery, Department of Surgery, Faculty of Medicine, Chiang Mai University, Chiang Mai, Thailand

**Keywords:** Acute heart failure, 30-day mortality, Hazard ratio, Emergency department, Risk factor

## Abstract

**Background:**

Acute heart failure is common in emergency departments, with medical technology promoting longer survival. Despite improvements in medical care, optimal criteria for discharge or admission decisions remain challenging to establish, particularly in resource-limited emergency department (ED) settings.

**Objective:**

To identify factors associated with 30-day all-cause mortality in patients with acute heart failure.

**Methods:**

This study was a retrospective cohort study conducted in Maharaj Nakorn Chiang Mai Hospital from 2011 to 2018. All patients diagnosed with acute de novo or decompensated heart failure and presenting to the ED were divided into two groups: those who died within 30 days and those who survived beyond 30 days. Factors obtained from medical history taking, physical examination, and laboratory results were compared between the two groups. Cox proportional hazards models were used to determine hazard ratios (HR) and 95% confidence intervals (95% CI).

**Results:**

We analyzed 1,951 patients diagnosed with acute de novo or decompensated heart failure. The overall 30-day all-cause mortality rate was 11.0%. Multivariable analysis identified seven independent prognostic factors. Six factors were associated with increased 30-day all-cause mortality: respiratory rate >30 breaths/min; HR 1.61 (95% CI 1.03–2.53), systolic blood pressure <90 mmHg; HR 2.25 (95% CI 1.13–4.45), creatinine levels >2 mg/dL; HR 1.61 (95% CI 1.08–2.39), serum sodium levels <135 mEq/L; HR 1.70 (95% CI 1.24–2.34), potassium levels >5.0 mEq/L; HR 1.59 (95% CI 1.02–2.49), and the use of inotropic drugs; HR 2.89 (95% CI 1.50–5.56). Conversely, dyslipidemia was associated with lower 30-day all-cause mortality (HR 0.54, 95% CI 0.36–0.81).

**Conclusion:**

Independent predictors of 30-day all-cause mortality included respiratory rate >30 breaths/min, SBP < 90 mmHg, creatinine >2 mg/dL, sodium <135 mEq/L, potassium >5.0 mEq/L, and inotrope use. These factors help identify high-risk patients requiring close monitoring and potential admission.

**Supplementary Information:**

The online version contains supplementary material available at 10.1186/s12872-025-05430-z.

## Introduction

Acute heart failure (AHF) is a complex syndrome with a concerning rise in incidence, high mortality, and morbidity [1]. Its prevalence varies depending on the definition, but it is about 1–2% in developed countries and over 10% among people over 70 years [2]. 

A study in Thailand found that Thai patients hospitalized for heart failure are younger and sicker than European and American patients. Heart failure with preserved ejection fraction is prevalent. The mortality rate was 5.4%, higher in those with poor LV systolic function [3]. 

Symptoms and signs of heart failure are often non-specific and may be mistaken for other problems. Breathlessness, orthopnea, and paroxysmal nocturnal dyspnea are typical symptoms [4]. Elevated jugular venous pressure, hepatojugular reflux, a third heart sound, and a laterally displaced apical impulse are more specific signs [5]. 

The evaluation of heart failure involves history taking, physical examination, and various investigations like chest X-ray, ECG, plasma NT-proBNP, echocardiography, CT, and angiography [6]. These methods require expertise and substantial resources. In rural hospitals, access to certain tests like plasma NT-proBNP, echocardiography, and CT is limited. Recent decades have seen the development of prognostic models using multiple markers to predict death or heart failure hospitalization [7–12]. However, their utility is limited by complexity and resource constraints. This study aims to identify prognostic factors that can be applied in rural or resource-limited emergency departments, derived from history taking, physical examination, and basic laboratory tests. These factors could enhance admission management for acute heart failure patients.

## Materials and methods

This retrospective cohort study adhered to the methodologic standards for medical record review outlined by Worster et al. (2005) [13] with ethics approval from the Research Ethics Committee, Faculty of Medicine, Chiang Mai University (STUDY CODE: EME-2564–08511).

### Participants

Patients aged ≥f18 years diagnosed with acute de novo heart failure and acute decompensated heart failure who presented to the emergency department (ED) at Maharaj Nakorn Chiang Mai Hospital from January 2011 to December 2018. The diagnosis and classification of heart failure were established in accordance with the 2021 ESC Guidelines. Acute de novo heart failure was defined as the onset of signs and symptoms of heart failure in a patient with no prior history of cardiac dysfunction. Conversely, acute decompensated heart failure (ADHF) was defined as the acute or gradual worsening of signs and symptoms in a patient with a previously established diagnosis of chronic heart failure. Patients were identified using ICD-10 codes for heart failure (I50.x) as the primary discharge diagnosis. The diagnosis was clinically adjudicated by the attending physicians based on the 2021 ESC Guidelines, incorporating clinical presentation, biomarkers, and imaging available during the ED visit. Patients presenting with cardiogenic shock (defined by hypotension requiring inotropic support) were included in this cohort to reflect the full spectrum of acute heart failure presentations in the emergency setting. Clear inclusion and exclusion criteria were applied, excluding patients who died from trauma.

### Outcome

To identify factors associated with 30-day all-cause mortality in Emergency Department Patients with Acute Heart Failure. The primary outcome was defined as 30-day all-cause mortality. Survival status was ascertained by linking patients’ hospital identification numbers with the Thai National Civil Registry database, which records all deaths nationwide, ensuring complete follow-up regardless of the location of death.

###  Predictors and pre-specified key predictors

Data were collected using a standardized data abstraction form with clearly defined variables. Trained abstractors conducted data extraction while blinded to the study hypothesis to minimize bias. Interobserver reliability was ensured by independent abstraction from two reviewers, with discrepancies resolved by consensus. To further ensure accuracy, abstractors underwent training before data collection, and their performance was periodically monitored throughout the process.

Variables included demographics (sex, age, and weight) and comorbidities (Chronic obstructive pulmonary disease (COPD), Cerebrovascular accident (CVA), cancer, and dementia). Vital signs (blood pressure, pulse rate, O2 saturation, and respiratory rate) were obtained from the initial assessment recorded by triage nurses upon the patient’s arrival, adhering to standard hospital protocols. Clinical symptoms (dyspnea, syncope, palpitation, fatigue, orthopnea, paroxysmal nocturnal dyspnea, chest pain, and ankle swelling) and physical examination findings (crepitation, wheezing, jugular venous engorgement, and peripheral edema) were extracted from the admission notes documented by the attending emergency physicians. Laboratory parameters (hemoglobin, BUN, creatinine, sodium, potassium, chloride, and bicarbonate) were obtained from the initial blood samples collected during the ED visit.

Key exposure definitions and clinical thresholds were pre-specified as follows:

- Demographics: Elderly status was defined as age >65 years.
- Hemodynamics and Vital Signs:
  - Systolic Blood Pressure (SBP): Hypotension was defined as SBP < 90 mmHg (chosen over MAP for bedside applicability) [14, 15].
  - Heart Rate (HR): Stratified into three categories: < 60 bpm, 60–100 bpm, and >100 bpm.
  - Respiratory Rate (RR): Categorized as < 21, 21–30, and >30 breaths/min [16].
  - Oxygen Saturation: Dichotomized as < 90% versus **≥** 90%.
- Laboratory Parameters: Clinical thresholds for abnormal laboratory findings were defined as:
  - Anemia: Hemoglobin < 10 g/dL.
  - Renal Impairment: Creatinine >2 mg/dL [14, 17].
  - Electrolyte Imbalances: Hyponatremia was defined as Sodium < 135 mEq/L. Potassium levels were categorized as hypokalemia (< 3.5 mEq/L), normokalemia (3.5–5.0 mEq/L), and hyperkalemia (>5.0 mEq/L) [17].
  - Acid-Base Status: Low bicarbonate was defined as < 20 mEq/L.
- Clinical Management: Inotropic drug use.

### Sample size and statistical power

The study size was determined by the total number of eligible patients presenting to the emergency department during the study period (January 2011 to December 2018). Consequently, a convenience sampling method was employed to include all available cases, resulting in a final cohort of 1,951 patients with 215 recorded 30-day all-cause mortality events.

To assess the robustness of the multivariable Cox regression model, we evaluated the events-per-variable (EPV) ratio. With 215 mortality events and 35 candidate predictors included in the full model, the calculated EPV was approximately 6.1. While this falls below the traditional rule of thumb of 10 events per variable, simulation studies by Vittinghoff and McCulloch have demonstrated that an EPV of 5–9 can still yield reliable estimates and valid confidence intervals in survival analysis contexts [18]. 

### Missing data

A consecutive sampling method was employed to reduce selection bias, and missing data were handled using a complete case analysis approach. The source of the medical records was clearly identified, and statistical management of missing data was predefined. Given the very low proportion of missing data in key predictors (< 5%), we did not perform multiple imputation, as the potential gain in precision would be minimal.

### Statistical analysis

Statistical analysis was conducted using STATA 16.0 (StataCorp, College Station, TX, USA). Outliers were excluded prior to analysis. Continuous variables were tested for normality using the Shapiro-Wilk test. Normally distributed data are presented as mean and standard deviation (SD) and compared using the independent t-test. Non-normally distributed data are presented as median and interquartile range (IQR) and compared using the Mann-Whitney U test. Categorical variables are expressed as frequencies and percentages and compared using Fisher’s exact test as appropriate. Results are presented as hazard ratios (HR) with 95% confidence intervals (95% CI). A multivariable Cox regression model was used to identify independent prognostic factors for 30-day all-cause mortality. All variables from the univariable analysis were included in the multivariable model, regardless of their significance level. These variables included: Age > 65 years, Sex (Male), Heart rate (< 60, 60–100, >100 bpm), Respiratory rate (< 21, 21–30, >30 breaths/min), Oxygen saturation (< 90% vs. ≥ 90%), Systolic BP < 90 mmHg, COPD, Asthma, Hypertension, Diabetes mellitus, Dyslipidemia, Atrial fibrillation, Chronic kidney disease, Cirrhosis, Cerebrovascular accident, Cancer, Dementia, Dyspnea, Syncope, Palpitation, Fatigue, Orthopnea, Paroxysmal nocturnal dyspnea, Chest pain, Ankle swelling, Jugular venous distension, Lung crepitation, Wheezing, S_3_/S_4_ gallop, Hemoglobin < 10 g/dL, Creatinine > 2 mg/dL, Sodium < 135 mEq/L, Potassium (< 3.5, 3.5–5.0.5.0, >5.0 mEq/L), Bicarbonate < 20 mEq/L, and Inotrope use. A p-value less than 0.05 was set as the significance level. A multivariable Cox regression model was used to identify independent prognostic factors. The validity of the model assumptions was rigorously checked. The proportional hazards assumption was verified using Schoenfeld residuals, with the global test yielding a p-value of 0.336, indicating no violations. Multicollinearity was assessed using the Variance Inflation Factor (VIF), resulting in a mean VIF of 1.29, which confirms the absence of significant collinearity among the predictors.

## Result

**Figure 1:**
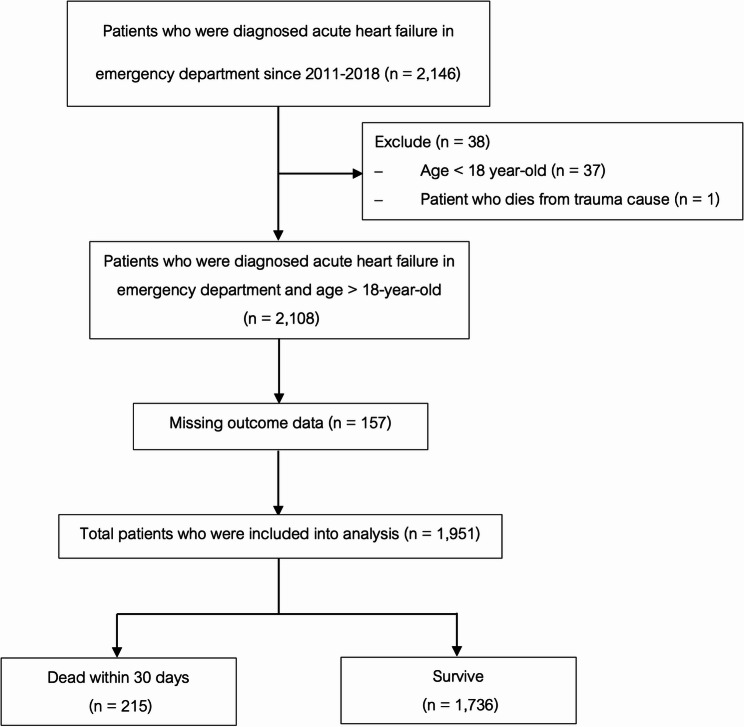
Study flow diagram

Out of 2,146 patients diagnosed with acute heart failure, 47 were excluded due to being under 18 and dying from other causes. Incomplete medical records were found for 157 patients, leaving 1,951 included in the study. Of these, 215 died within 30 days and 1,736 survived. The overall 30-day all-cause mortality rate was 11.0% (as shown in Figure. 1 and Figure S1).

Analysis of baseline characteristics revealed significant differences in various factors, including hypertension, diabetes, dyslipidemia, cancer, palpitation, nocturnal dyspnea, chest pain, blood pressure, urea nitrogen, creatinine, sodium, potassium, chloride, and inotropic drug use. (Details are shown in Table 1)

**Table 1:** Baseline characteristics of acute de Novo and acute decompensated heart failure patients (*n* = 1,951)

Characteristics	Missing *N* (%)	Dead (*n* = 215)	Alive (*n* = 1,736)	*p*-value
Patient factors				
Male*	0 (0)	103 (47.9)	815 (46.9)	0.828
Age (years)**	0 (0)	68.44 (16.1)	67.69 (15.0)	0.494
Co-morbidity*				
COPD	0 (0)	25 (11.6)	113 (6.5)	0.010
Asthma	0 (0)	8 (3.7)	49 (2.8)	0.397
Hypertension	0 (0)	103 (47.9)	1,051 (60.5)	0.001
Diabetes mellitus	0 (0)	56 (26.0)	582 (33.5)	0.031
Dyslipidemia	0 (0)	47 (21.9)	597 (34.4)	< 0.001
Prior myocardial infarction	0 (0)	99 (46.0)	726 (41.8)	0.242
Atrial fibrillation	2 (0.1)	50 (23.3)	332 (19.2)	0.171
Chronic kidney disease	1 (0.05)	64 (29.8)	480 (27.7)	0.520
Cirrhosis	0 (0)	7 (3.3)	28 (1.6)	0.098
Cerebrovascular accident (CVA)	1 (0.05)	21 (9.8)	127 (7.3)	0.217
Cancer	0 (0)	17 (7.9)	63 (3.6)	0.006
Dementia	0 (0)	5 (2.3)	15 (0.9)	0.060
Symptoms*				
Dyspnea/Breathlessness	1 (0.05)	197 (91.6)	1,553 (89.5)	0.404
Syncope	1 (0.05)	2 (0.9)	38 (2.2)	0.308
Palpitation	1 (0.05)	17 (7.9)	237 (13.7)	0.018
Fatigue	1 (0.05)	21 (9.8)	112 (6.5)	0.084
Orthopnea	2 (0.1)	120 (55.8)	974 (56.2)	0.942
Paroxysmal nocturnal dyspnea	3 (0.15)	74 (34.6)	725 (41.8)	0.047
Chest pain	1 (0.05)	40 (18.6)	504 (29.0)	0.001
Ankle swelling	4 (0.2)	121 (56.5)	952 (54.9)	0.663
Signs				
O_2_ saturation (%) **	80 (4.10)	89.32 (9.9)	90.54 (9.9)	0.098
Systolic BP (mmHg.) **	6 (0.31)	123.4 (30.5)	142.54 (32.3)	< 0.001
Diastolic BP (mmHg.) **	6 (0.31)	72.5 (20.1)	80.53 (20.8)	< 0.001
Pulse rate (beat per minute) **	5 (0.26)	92.96 (25.8)	91.42 (23.2)	0.364
Temperature (Celsius) **	435 (22.3)	36.76 (0.8)	36.70 (0.7)	0.256
Wheezing *	1 (0.05)	31 (14.4)	191 (11.0)	0.139
Jugular venous distension *	9 (0.46)	40 (18.7)	283 (16.4)	0.382
Pulmonary crepitations *	1 (0.05)	172 (80.0)	1,385 (79.8)	1.000
Investigations				
Hemoglobin (g/dL) **	66 (3.38)	11.1 (2.5)	11.3 (2.6)	0.384
Hematocrit (%) **	67 (3.43)	34.6 (7.7)	35.2 (7.8)	0.349
B.U.N. (mg/dL) ***	70 (3.59)	31 (21–52.5.5)	23 (15–38)	< 0.001
Creatinine (mg/dL) ***	65 (3.33)	1.7 (1.1–3.2)	1.35 (1.0–2.4.0.4)	< 0.001
Serum sodium (mEq/L) **	64 (3.28)	135.63 (6.8)	137.56 (5.5)	< 0.001
Serum potassium (mEq/L) **	63 (3.23)	4.25 (0.8)	4.07 (0.7)	< 0.001
Serum chloride (mEq/L) **	64 (3.28)	97.93 (7.4)	101.13 (7.1)	< 0.001
Serum bicarbonate (mEq/L) **	67 (3.43)	21.89 (5.9)	22.51 (4.7)	0.080
Clinical management				
Inotrope *	11 (0.56)	22 (10.3)	32 (1.8)	< 0.001

*p*-values were calculated using Fisher’s exact test for categorical variables, and the independent t-test or Mann-Whitney U test for continuous variables

* Number (percentage)

** mean (standard deviation)

*** median (interquartile range)

*COPD* Chronic obstructive pulmonary disease, *B.U.N.* Blood urea nitrogen

Calculating the univariable hazard ratio reveals that respiratory rate of 30 breaths per minute, oxygen saturation below 90% on room air, and systolic blood pressure below 90 mmHg increase the mortality rate within the vital signs group. Chronic obstructive pulmonary disease (COPD), cancer, and dementia increase mortality rates, while hypertension, diabetes, and dyslipidemia decrease them. Palpitation and paroxysmal nocturnal dyspnea are more prevalent in the deceased group. In the basic laboratory investigation group, creatinine levels above 2 mg/dL, sodium levels below 135 mEq/L, potassium levels above 5.0 mEq/L, and bicarbonate levels below 20 mEq/L increase mortality rates. Patients requiring inotropic drugs have a higher mortality rate.

Multivariable analysis identified seven independent prognostic factors. Six factors were associated with increased 30-day all-cause mortality: respiratory rate > 30/min (HR 1.61, 95% CI 1.03–2.53), systolic blood pressure < 90 mmHg (HR 2.25, 95% CI 1.13–4.45), creatinine > 2 mg/dL (HR 1.61, 95% CI 1.08–2.39), sodium < 135 mEq/L (HR 1.70, 95% CI 1.24–2.34), potassium > 5.0 mEq/L (HR 1.59, 95% CI 1.02–2.49), and inotrope use (HR 2.89, 95% CI 1.50–5.56) (Table 2 and Figure S2). Conversely, dyslipidemia was associated with lower 30-day all-cause mortality (HR 0.54, 95% CI 0.36–0.81).

**Table 2:** Univariable and multivariable hazard ratio of 30-day all-cause mortality in emergency department

Characteristics (*n* = 1,723)	Univariable hazard ratio (95% CI)	*p*-value	Multivariable hazard ratio (95% CI) *	*p*-value
Age, (year)	1.00 (0.99–1.01)	0.420		
Age > 65 years	1.15 (0.86–1.53)	0.339	1.16 (0.82–1.63)	0.401
Sex (Male)	1.02 (0.77–1.34)	0.889	0.97 (0.71–1.32)	0.842
Heart rate	1.00 (0.99–1.01)	0.567		
HR < 60	1.33 (0.76–2.31)	0.312	1.06 (0.56–2.00.56.00)	0.851
HR 60–100	Reference		-	
HR > 100	1.04 (0.77–1.40)	0.814	0.81 (0.57–1.15)	0.243
Respiratory rate	1.03 (1.01–1.05)	< 0.001		
RR < 21	Reference		-	
RR 21–30	1.32 (0.91–1.90)	0.143	0.96 (0.64–1.45)	0.855
RR > 30	1.90 (1.28–2.82)	0.001	1.613 (1.03–2.53)	0.037
Oxygen saturation	0.99 (0.98–1.00.98.00)	0.087		
≥ 90%	Reference		-	
< 90%	1.46 (1.09–1.95)	0.011	1.26 (0.91–1.74)	0.167
Systolic BP (mmHg)	0.98 (0.97–0.99)	< 0.001		
Systolic BP (< 90 mmHg.)	3.45 (1.96–6.05)	< 0.001	2.25 (1.13–4.45)	0.020
Comorbidity				
COPD	1.800 (1.17–2.76)	0.007	1.56 (0.93–2.62)	0.089
Asthma	1.36 (0.67–2.77)	0.388	1.52 (0.72–3.21)	0.271
Hypertension	0.62 (0.47–0.81)	0.001	0.77 (0.54–1.11)	0.162
Diabetes mellitus	0.69 (0.51–0.95)	0.023	0.90 (0.62–1.33)	0.611
Dyslipidemia	0.54 (0.39–0.75)	< 0.001	0.54 (0.36–0.81)	0.003
Prior myocardial infarction	1.06 (0.80–1.39)	0.699	1.13 (0.83–1.54)	0.443
Atrial fibrillation	1.27 (0.92–1.76)	0.145	1.21 (0.84–1.73)	0.311
Chronic kidney disease	1.11 (0.82–1.50)	0.481	0.74 (0.50–1.11)	0.150
Cirrhosis	1.95 (0.92–4.15)	0.082	1.95 (0.89–4.27)	0.094
Cerebrovascular accident	1.42 (0.90–2.23)	0.130	1.20 (0.69–2.09)	0.510
Cancer	2.06 (1.23–3.43)	0.006	1.77 (0.98–3.18)	0.056
Dementia	2.66 (1.10–6.47)	0.031	1.90 (0.57–6.35)	0.294
Symptoms				
Dyspnea	1.35 (0.81–2.25)	0.249	1.04 (0.55–1.98)	0.894
Syncope	0.23 (0.03–1.66)	0.146	0.39 (0.05–2.97)	0.365
Palpitation	0.52 (0.31–0.88)	0.014	0.67 (0.38–1.21)	0.185
Fatigue	1.56 (0.98–2.47)	0.060	0.98 (0.55–1.76)	0.952
Orthopnea	0.99 (0.75–1.30)	0.933	1.20 (0.84–1.71)	0.304
Paroxysmal nocturnal dyspnea	0.77 (0.58–1.03)	0.081	0.72 (0.51–1.01)	0.059
Chest pain	0.54 (0.38–0.77)	0.001	0.68 (0.45–1.02)	0.063
Ankle swelling	1.13 (0.85–1.49)	0.392	1.12 (0.81–1.54)	0.502
Signs				
Jugular venous distension	1.17 (0.82–1.66)	0.393	1.32 (0.90–1.94)	0.160
Lung crepitation	1.03 (0.73–1.45)	0.877	0.99 (0.66–1.50)	0.978
Wheezing	1.35 (0.92–1.99)	0.125	1.19 (0.75–1.88)	0.453
S_3_S_4_ gallop	0.52 (0.24–1.14)	0.104	0.45 (0.18–1.09)	0.078
Hemoglobin	0.98 (0.92–1.03)	0.367		
Hemoglobin < 10 g/dL.	1.21 (0.91–1.62)	0.195	1.10 (0.78–1.54)	0.587
Creatinine	1.04 (1.00–1.08.00.08)	0.051		
Creatinine > 2 mg/dL.	1.72 (1.30–2.28)	< 0.001	1.61 (1.08–2.39)	0.019
Sodium	0.95 (0.93–0.97)	< 0.001		
Sodium < 135 mEq/L.	2.13 (1.60–2.84)	< 0.001	1.70 (1.24–2.34)	0.001
Potassium	1.34 (1.13–1.59)	0.001		
Potassium < 3.5 mEq/L.	1.14 (0.79–1.66)	0.487	1.23 (0.82–1.85)	0.306
Potassium 3.5–5.5 mEq/L	Reference			
Potassium > 5.0 mEq/L.	2.02 (1.38–2.98)	< 0.001	1.59 (1.02–2.49)	0.041
Bicarbonate	0.977 (0.95–1.01)	0.122		
Bicarbonate < 20 mEq/L.	1.47 (1.09–1.98)	0.011	1.06 (0.74–1.52)	0.741
Inotrope use	4.93 (3.11–7.83)	< 0.001	2.89 (1.50–5.56)	0.002

*Completed case analysis (*n* = 1,723)

*COPD* Chronic obstructive pulmonary disease

The performance of the final multivariable Cox regression model was evaluated for discrimination and calibration. The model demonstrated good discrimination with a Harrell’s C-index of 0.71. Calibration was assessed using the Gronnesby and Borgan goodness-of-fit test, which yielded a p-value of 0.48, indicating no significant deviation from the model assumptions and suggesting an adequate fit.

## Discussion

From the analyzed data, increased respiratory rate (>30/min.) is often associated with pulmonary pathology, impairing oxygen and carbon dioxide exchange. To enhance minute ventilation, the body increases its respiratory rate. If treatment is ineffective, anaerobic metabolism occurs, leading to acidosis, shock, and death [19]. 

Hypotension, especially systolic blood pressure < 90 mmHg, is a critical sign of hemodynamic compromise. Low blood pressure reduces systemic perfusion, leading to tissue hypoxia and shock [20]. Regarding inotrope use, we interpret this factor as a surrogate marker of disease severity rather than a direct causal mechanism. The requirement for inotropic support identifies a subgroup of patients with profound hemodynamic instability or cardiogenic shock. Therefore, the strong association with mortality (HR 2.89) reflects the critical condition of patients requiring such intervention, serving as a proxy for acute circulatory failure. We acknowledge the potential for indication bias; however, the requirement for inotropes effectively identifies a subgroup of patients with critically low cardiac output and imminent mortality risk. This may explains why these hemodynamic markers (systolic blood pressure < 90 mmHg and inotrope use) are strongly associated with the 30-day all-cause mortality rate.

Blood test results associated with the 30-day all-cause mortality rate include creatinine >2 mg/dL, sodium levels < 135 mEq/L, and potassium levels >5.0 mEq/L. These results often reflect complications from shock, which reduces blood flow to vital organs, including the kidneys. Impaired kidney function leads to increased creatinine levels, a widely used marker for kidney function [21]. Elevated creatinine levels may indicate acute kidney injury, which contributes to increased mortality rates. Decreased kidney function leads to elevated blood potassium levels, which depolarize cardiac cells and make them more excitable. This can cause premature depolarizations, arrhythmias such as premature atrial contractions (PACs) or premature ventricular contractions (PVCs), and in severe cases, life-threatening arrhythmias such as ventricular tachycardia or ventricular fibrillation [22]. These arrhythmias can result in sudden cardiac arrest and require immediate medical intervention.

Hyponatremia in acute heart failure occurs due to decreased cardiac output, reduced kidney perfusion, and activation of the renin-angiotensin-aldosterone system (RAAS) and sympathetic nervous system, causing sodium and water retention. If cardiac output remains low, renal perfusion may be compromised, leading to decreased water excretion and further hyponatremia. Antidiuretic hormone (ADH) also contributes to hyponatremia in acute heart failure. ADH release increases blood volume and water reabsorption in the kidneys, causing water retention and dilutional hyponatremia [23]. Therefore, hyponatremia is associated with the development of volume overload, which can lead to acute pulmonary edema and death.

Interestingly, our multivariable analysis revealed that dyslipidemia was associated with a significantly lower risk of 30-day all-cause mortality (HR 0.54). This finding aligns with the phenomenon known as ‘reverse epidemiology’ or the ‘lipid paradox’ frequently observed in heart failure patients, where higher lipid levels are paradoxically associated with better survival outcomes [24, 25]. Several mechanisms may explain this observation. First, higher lipoprotein levels may bind and neutralize circulating endotoxins, reducing inflammation [26]. Second, low cholesterol levels in heart failure often reflect a state of malnutrition, cachexia, and advanced disease severity, which are strong predictors of poor prognosis [24, 27]. Additionally, the diagnosis of dyslipidemia likely serves as a proxy for the prescription of statins. Although our database lacked specific medication records, statins have established pleiotropic effects—including anti-inflammatory and endothelial stabilizing properties—that may confer a short-term survival benefit [28]. However, given the retrospective nature of this study and the absence of specific pharmacological data (specifically statin therapy), we cannot rule out confounding by indication. Therefore, these interpretations regarding the protective role of dyslipidemia should be considered hypothesis-generating rather than definitive causal associations. Although this study was conducted in a tertiary university hospital, we deliberately focused on variables that are universally available in resource-limited or primary care settings (e.g., vital signs and basic metabolic panels), avoiding reliance on advanced biomarkers or imaging (e.g., NT-proBNP or Echocardiography). This design choice ensures that the identified risk factors apply to physicians in rural emergency departments, bridging the gap between tertiary-level findings and primary care application.

This study has several limitations. Firstly, this was a retrospective cohort study with missing data, with limited the inclusion of some variables for analysis. Secondly, the study included individuals from a single tertiary hospital in northern Thailand, , which may limit generalizability. Thirdly, the causes of death within 30 days were not documented, which could have identified other relevant factors. Certain factors (like dyslipidemia) lacked data to explain the observed outcomes, deviating from the initial hypothesis. Future prospective studies incorporating detailed pharmacological records (to account for statin use) and nutritional status indicators (e.g., albumin, BMI) are necessary to validate the mechanism behind the protective dyslipidemia paradox observed in this cohort. Finally, although the predictor variables were pre-specified according to the study protocol, the number of included variables relative to the number of outcome events (215 deaths) resulted in a low events-per-variable (EPV) ratio. Readers should interpret the multivariable analysis with caution due to the potential risk of model overfitting.

## Conclusion

Independent predictors of increased 30-day all-cause mortality included respiratory rate > 30/min, systolic blood pressure < 90 mmHg, creatinine > 2 mg/dL, sodium < 135 mEq/L, potassium > 5.0 mEq/L, and inotrope use. In contrast, dyslipidemia was independently associated with lower 30-day all-cause mortality in this cohort. This association is likely influenced by underlying patient characteristics and requires confirmation in future studies.

## Supplementary Information

Supplementary Material 1. Figure S1: Kaplan-Meier curve demonstrates the time-to-death.

Supplementary Material 2. Figure S2: Kaplan-Meier survival curves for 30-day all-cause mortality stratified by independent predictors. The curves demonstrate survival probability over 30 days stratified by: (A) Systolic Blood Pressure, (B) Creatinine, (C).

## Data Availability

The datasets are available from the corresponding author upon reasonable request.
