# Supplementary material for: Factors associated with 30-day mortality in patients with acute heart failure presenting to the emergency department: a retrospective cohort study
Source: BMC Cardiovasc Disord. 2025 Dec 12;26:44. doi: 10.1186/s12872-025-05430-z (PMC12809968; doi:10.1186/s12872-025-05430-z)
Supplement: Supplementary file 1 — Supplementary Material 1. Figure S1: Kaplan-Meier curve demonstrates the time-to-death. [file 12872_2025_5430_MOESM1_ESM.pdf]

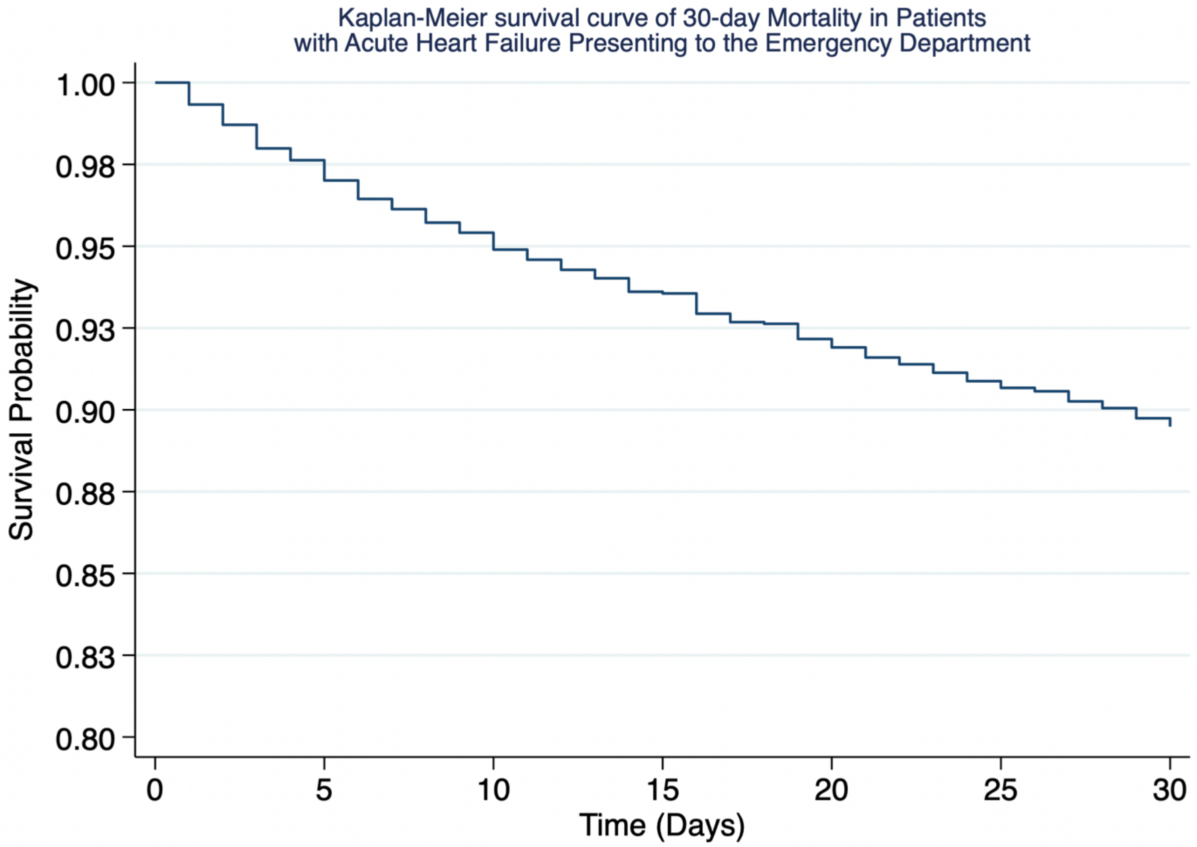
Kaplan-Meier survival curve of 30-day Mortality in Patients
with Acute Heart Failure Presenting to the Emergency Department
Survival Probability
1.00
0.98
0.95
0.93
0.90
0.88
0.85
0.83
0.80
0
5
10
15
20
25
30
Time (Days)
