# Supplementary figures and images for: Factors associated with 30-day mortality in patients with acute heart failure presenting to the emergency department: a retrospective cohort study
Source: BMC Cardiovasc Disord. 2025 Dec 12;26:44. doi: 10.1186/s12872-025-05430-z (PMC12809968; doi:10.1186/s12872-025-05430-z)

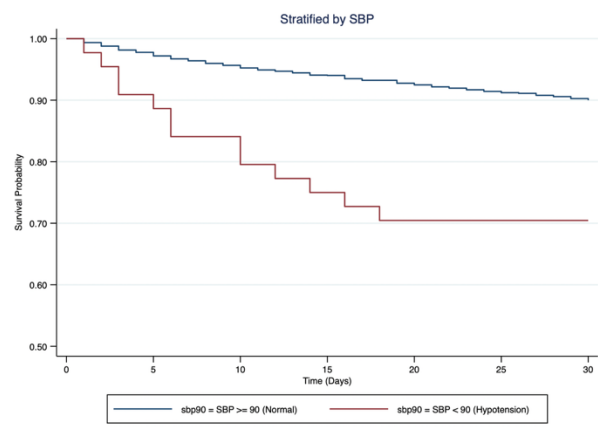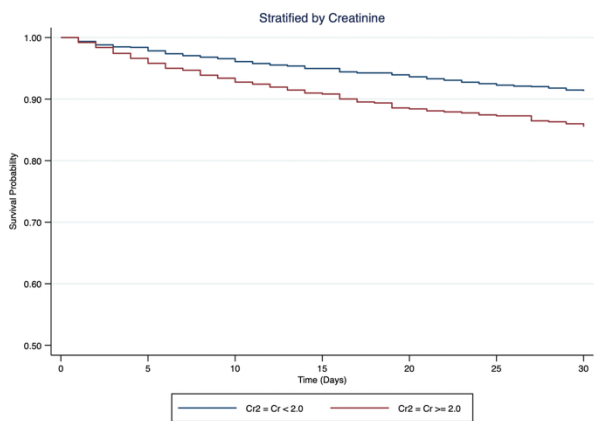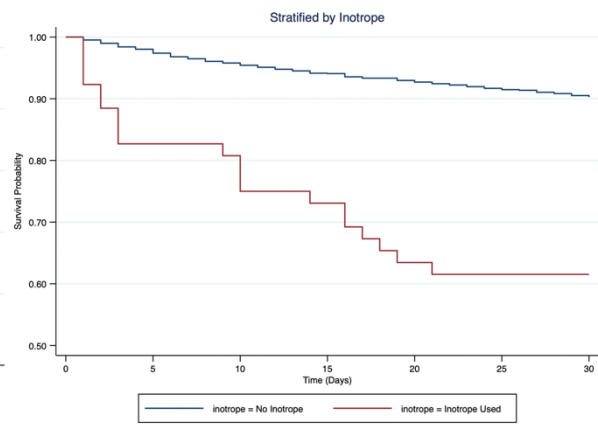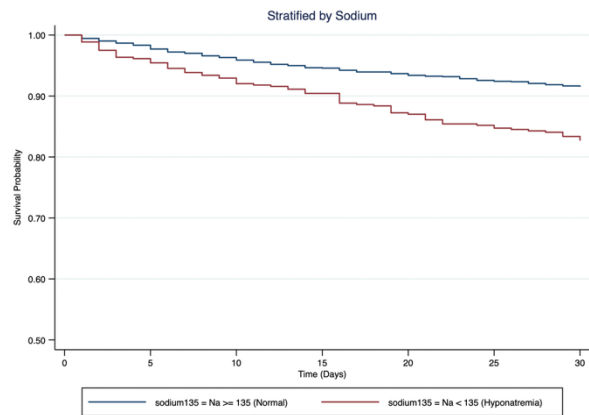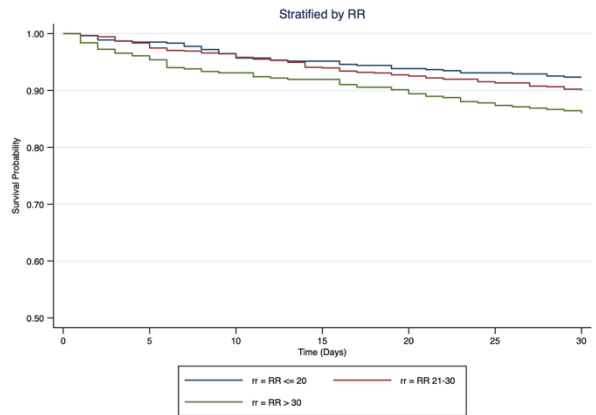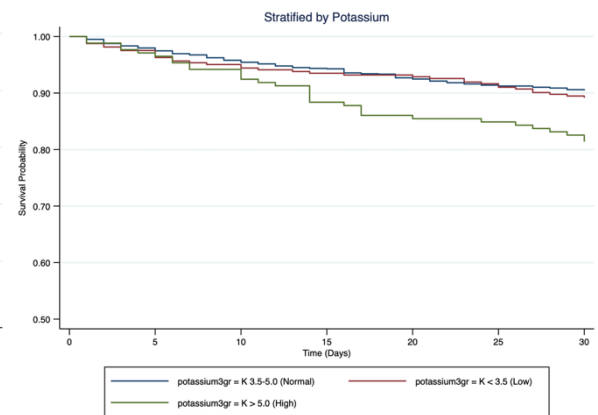

Supplement: Supplementary file 2 — Supplementary Material 2. Figure S2: Kaplan-Meier survival curves for 30-day all-cause mortality stratified by independent predictors. The curves demonstrate survival probability over 30 days stratified by: (A) Systolic Blood Pressure, (B) Creatinine, (C). [file 12872_2025_5430_MOESM2_ESM.pdf]
